# The effects of berberine on hyperhomocysteinemia and hyperlipidemia in rats fed with a long-term high-fat diet

**DOI:** 10.1186/1476-511X-11-86

**Published:** 2012-07-04

**Authors:** Xin-xia Chang, Hong-mei Yan, Qiong Xu, Ming-feng Xia, Hua Bian, Teng-fang Zhu, Xin Gao

**Affiliations:** 1 Department of Endocrinology and Metabolism, Zhongshan Hospital, Fudan University, Shanghai 200032, China; 2 Department of Pathology, Shanghai Medical College, Fudan University, Shanghai 200032, China

**Keywords:** Berberine, Hyperhomocysteinemia, Hyperlipidemia

## Abstract

**Background:**

The study was undertaken to examine the effects of berberine (BBR) on serum homocysteine, lipids and the aortic lesion in Sprague–Dawley (SD) rats fed with a long-term high-fat diet (HFD).

**Methods:**

Healthy male SD rats weighing 190-210 g received randomly standard diet or a high-fat diet for 24 weeks. After 8 weeks of feeding, rats fed with HFD were randomized to receive berberine (200 mg · kg-1· day-1) or vehicle by gavage for 16 weeks. After overnight fasting, all rats were sacrificed and total blood samples were also collected for determinant of fasting serum homocysteine (Hcy), total cholesterol (TC) and low density lipoprotein cholesterol (LDL-c) levels. The aorta was stained with hematoxylin and eosin (HE) and Sudan Ш to evaluate aortic lesion. The livers were dissected out and snap-frozen in liquid nitrogen for hepatic TC content and molecular analysis. 3-hydroxy-3-methyl-glutaryl-CoA reductase (HMGR), Lipoprotein receptors and apolipoproteins gene expression in the liver were determined by real-time PCR.

**Results:**

Intragastrical administration with berberine for 16 weeks lowered serum Hcy in rats fed with a high-fat diet. In parallel, it also decreased body weight and improved serum TC and LDL-c. Berberine also tended to decrease hepatic cholesterol. Consistently, berberine also upregulated LDL receptor (LDLR) mRNA level and suppressed HMGR gene expression. Meanwhile, upon berberine-treated rats, there was a significant increase in apolipoprotein E (apoE) mRNA, but no change in apoAI and scavenger receptor (SR) mRNA in the liver. Further, no atherosclerotic lesions were developed in berberine-treated rats for 16 weeks.

**Conclusion:**

Berberine can counteract HFD-elicited hyperhomocysteinemia and hyperlipidemia partially via upregulating LDLR and apoE mRNA levels and suppressing HMGR gene expression.

## Background

Homocysteine (Hcy) is a sulfur-containing amino acid formed during the metabolism of methionine. Hyperhomocysteinemia (HHcy) as a potent pro-inflammatory factor might accelerate the development of atherosclerosis [1]. Furthermore, the increased risk for vascular disease from elevated homocysteine is similar to that of other major cardiovascular risk factors. More importantly, it is independent of these factors [2-4]. Thus, reducing total Hcy levels can prevent the development of arterosclerotic vascular disease. But, now it remains unclear whether berberine affects the level of serum Hcy.

Berberine (BBR), a natural alkaloid extracted from *Coptis chinensis*, is previously used for diarrhea treatment. In 1986, Chen et al. [5] at first reported that berberine can lower serum glucose levels, besides anti-inflammatory. Many studies [6-11] subsequently demonstrated that berberine has also beneficial effects in the improvement of lipid and glucose metabolism. Weight loss, lowering serum low density lipoprotein cholesterol (LDL-c) and glucose can prevent the development of arteriosclerosis. Thus, these studies suggested that berberine have the potential effect on preventing the development of atherosclerosis for these beneficial metabolic effects.

However, it was recently reported that berberine promotes atherosclerosis development in apolipoprotein E-deficient (apoE^-/-^) mice, which could counter-balance the beneficial effect of lowering serum cholesterol [12]. ApoE^-/-^ mouse is a well-established animal model for studying atherosclerosis [13-15], but, the pathogenesis in these mice is strikingly different from that of human atherosclerosis. Thus, the potential of berberine for affecting the level of serum homocysteine and atherosclerosis needs to be more carefully investigated. This study will observe the effects of berberine on atherogenic factors of atherosclerosis (e.g. serum Hcy and lipids) and the aortic lesion in wild rats fed with a long-term high-fat diet.

## Results

### Berberine lowered serum hcy and improved typical risk factors related to atherosclerosis in rats fed with a high-fat diet (HFD)

**Figure 1:**
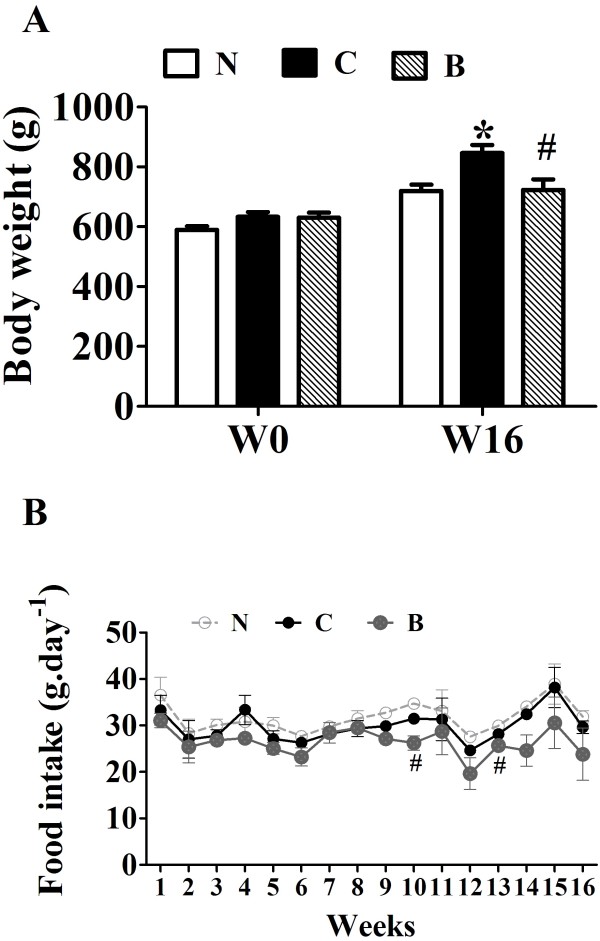
**Effects of berberine (BBR) on body weight and food intake of rats.** Male SD rats at 6 weeks of age received a high-fat diet (HFD) or regular rodent chow. After 8 weeks of feeding, rats were treated for 16 weeks with BBR or vehicle (n = 8 per group). **A**: The body weight is the average weight of 8 rats at the beginning of BBR treatment and after 16-week experiment. N, Normal control; C, vehicle –treated rats fed with HFD; B, rats with berberine treatment (200 mg/kg/d). **B**: Effects of BBR on food intake. The food intake was measured every week. Values are mean ± SEM. *p < 0.05*vs* N; ^#^*p* < 0.05 *vs* C.

The body weight of SD rats was gradually increased with a high-fat diet for 24 weeks. Body weights of HFD-fed rats were significantly reduced in BBR -treated group for 16 weeks (Figure 1A) as compared with control group with no dramatic changes in food intake (Figure 1B).

**Figure 2:**
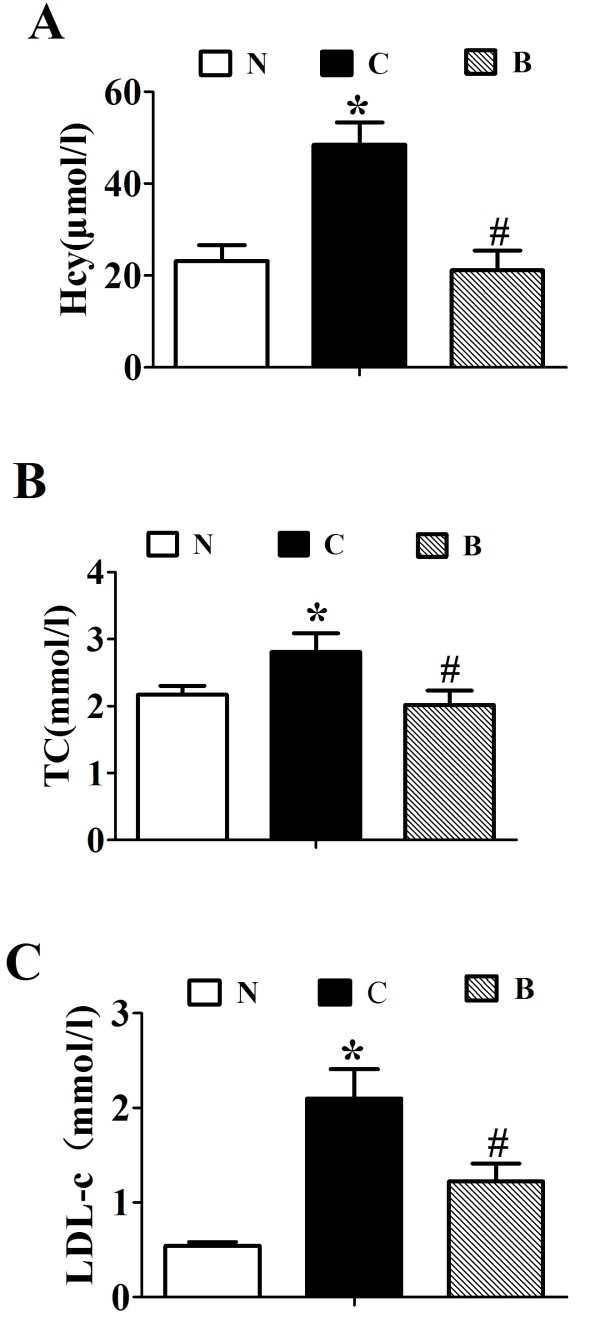
**Effects of berberine (BBR) on serum Hcy and lipid profile.** Serum Hcy (**A**), TC (**B**) and LDL-c (**C**) are the average of each group (n = 8). N, Normal control; C, vehicle –treated rats fed with HFD; B, rats with berberine treatment (200 mg/kg/d). Values are mean ± SEM. *p < 0.05*vs* N; ^#^*p* < 0.05 *vs* C.

Intragastrical administration with BBR for 16 weeks, serum homocysteine was significantly decreased by about 60% in contrast to the vehicle-treated rats fed with the same high-fat diet (*p* < 0.001, Figure 2A). BBR treatment significantly lowered the levels of serum TC (*p* < 0.05, Figure 2B) and LDL-c (*p* < 0.05, Figure 2C) as compared with the placebo group in SD rats fed with a high-fat diet. These data suggested that BBR exert antagonizing effects on HFD-induced dyslipidemia and hyperhomocysteinemia in SD rats.

### BBR decreased hepatic cholesterol in rats fed with HFD

**Figure 3:**
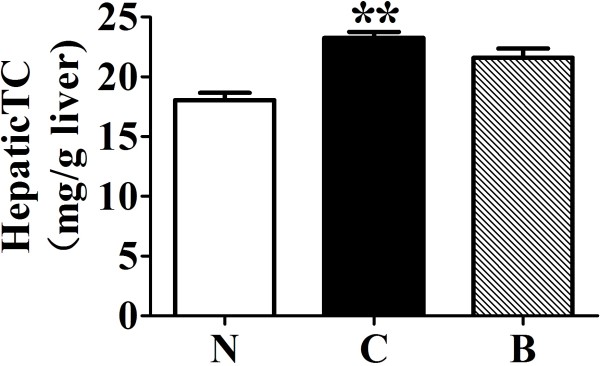
**Effects of berberine (BBR) on hepatic total cholesterol.** N, Normal control; C, vehicle –treated rats fed with HFD; B, rats with berberine treatment (200 mg/kg/d). Values are mean ± SEM. Significance was assessed by one-way ANOVA followed by Tukey's Multiple Comparison test. Data are mean ± SEM. *p < 0.05, **p < 0.01 *vs* N.

The effects of berberine on total cholesterol level were measured in rat liver. Rats fed with HFD alone showed a significant (*p* < 0.001) increase in hepatic cholesterol content, when compared with the normal controls (Figure 3). Treatment of high-fat diet-fed rats with berberine (200 mg/kg/d) tended to reduce hepatic cholesterol level, although this difference failed to reach statistical significance when compared to the high-fat diet control.

### Berberine reverses HFD-elicited abnormal expression of some key genes related to cholesterol metabolism in the liver

**Figure 4:**
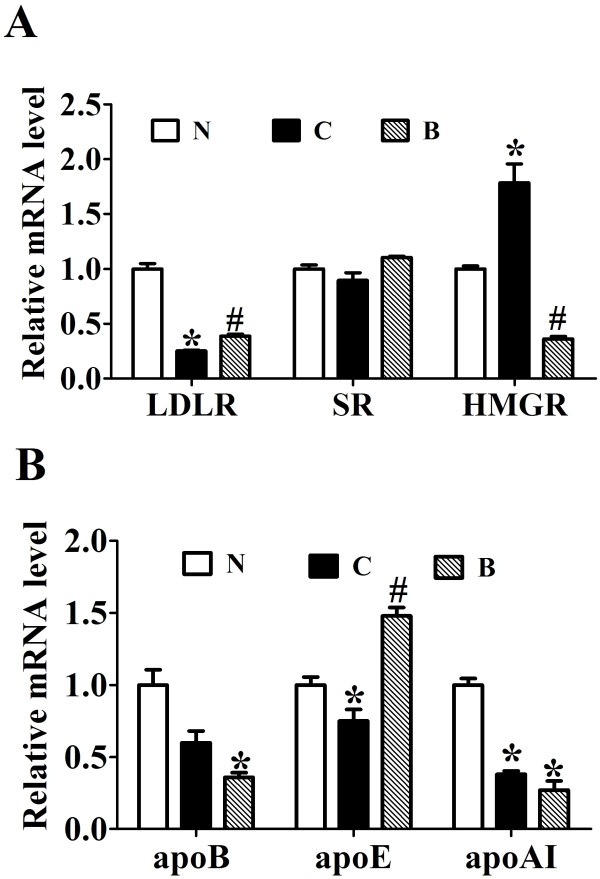
**The effects of berberine on 3-hydroxy-3-methyl-glutaryl-CoA reductase (HMGR), Lipoprotein receptors and apolipoproteins gene expression.** Real-time quantitative PCR analysis of LDLR, HMGR and SR (**A**) and apolipoproteins (**B**) in the livers of SD rats administrated with berberine or vehicle (n = 8 per group). Relative mRNA amounts of each gene were normalized to that of *beta-actin*. Values are mean ± SEM. *p < 0.05*vs* N; ^#^*p* < 0.05 *vs* C.

The effects of berberine on 3-hydroxy-3-methyl-glutaryl-CoA reductase (HMGR), lipoprotein receptors and apolipoproteins gene expression in the liver of high-fat diet-fed rats were investigated. The relative mRNA levels of low-density lipoprotein receptor (LDLR) (*p* < 0.05, Figure 4A) and apoE (*p* < 0.05, Figure 4B) was significantly down-regulated in the livers of HFD-fed rats relative to the ND control group. BBR treatment significantly reversed the downregulating effects of HFD on the expression of LDLR and apoE (*p* < 0.05). On the other hand, the level of HMGR mRNA was increased in rats fed with long-term high-fat diet, which was markedly lowered by BBR treatment (*p* < 0.05, Figure 4A). We also found that apoB and apoAI mRNAs were suppressed by HFD feeding (*p* < 0.05, Figure 4B), but these were not affected by BBR treatment. Despite these changes, BBR and high-fat-diet feeding did not influence the expression of scavenger receptor (SR).

### Effect of BBR treatment for 16 weeks on aortic lesions in rats fed with a high-fat diet

**Figure 5:**
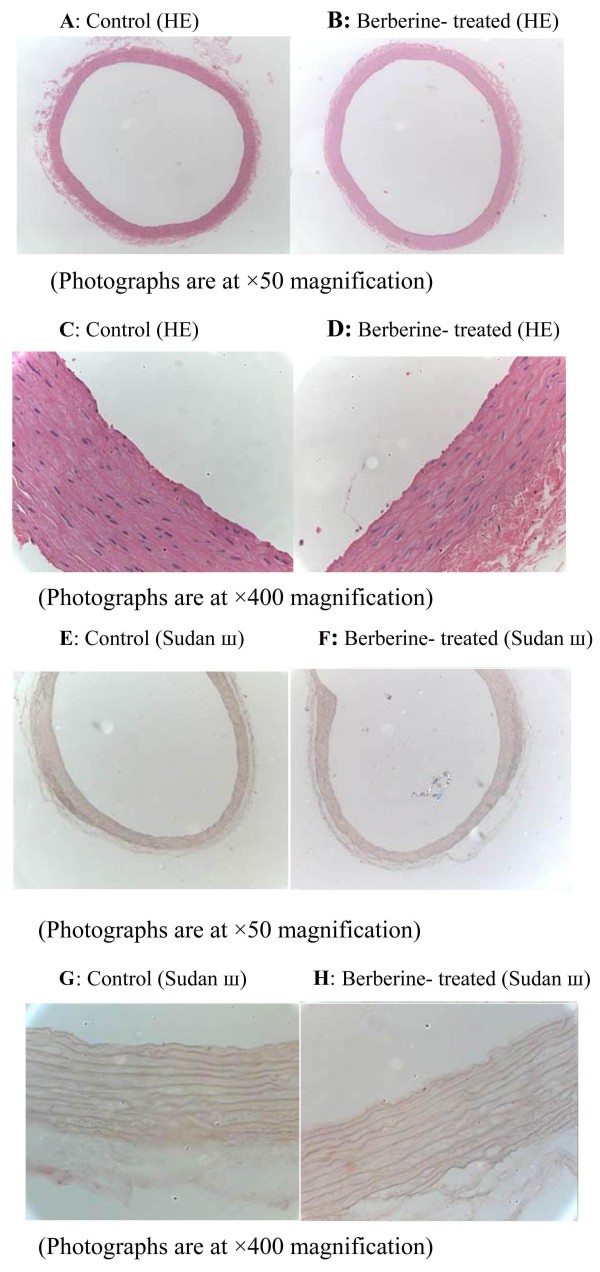
**Effects of berberine (BBR) on the aorta of rats fed with a high-fat diet.** After BBR treatment for 16 weeks, rats were killed after a 14-hour fast. Histological analysis of the aorta of the HFD-fed rats treated with vehicle (C group) or BBR (B group). The aorta sections were stained with hematoxylin and eosin (A-D) or with Sudan Ш (E-I) to evaluate the pathologic structures. Representative photographs are shown. **A-B, E-F:** Photographs are at × 50 magnification, **C-D, G-H:** Photographs are at × 400 magnification.

Histological analysis by HE staining of the aorta showed that the tunica intima of aorta remained smooth and intact in rats with a 24- weeks high-fat diet after treatment with berberine for 16 weeks (Figure 5A-D). The results of aorta stained with Sudan III showed that no lipid inclusions and foam cell accumulation were seen in the aortic endothelium of these rats (Figure 5E-H). Furthermore, in other rats fed with a high-fat diet without administration of berberine, there were also no deposits of monocyte-derived macrophages and abnormal fatty droplets in aortic tunica intima. These results suggested that no atherosclerotic lesions were developed in berberine-treated for 16 weeks and control rats fed with a long-term high-fat diet.

## Discussion

In this study, we have shown that berberine could decrease serum homocysteine level and many known risk factors related to atherosclerosis, such as body weight, serum cholesterol, and LDL-c in SD rats fed with a high-fat diet. Consistently berberine could upregulate LDLR and apoE, and downregulate HMGR gene expression in the liver. Importantly, our morphological results demonstrated that berberine do not increase the development of atherosclerosis in SD rats fed with a long-term high-fat diet.

Recent studies demonstrate that hyperhomocysteinemia (HHcy) as a potent pro-inflammatory factor increases the risk for the development of atherosclerosis, which is independent of these typical cardiovascular risk factors [1-4]. The results of a meta-analysis [16] including 27 studies relating homocysteine to arteriosclerotic vascular disease and 11 studies of folic acid effects on serum homocysteine levels population-based case–control studies showed that a 5-mu mol/L total Hcy (tHcy) increment causes a 1.6-fold and a 1.8-fold increase in risk for coronary artery disease (CAD) for men and women, respectively. Further, a total of 10% of the population's CAD risk appears attributable to tHcy. The OR for cerebrovascular disease (5-mu mol/L tHcy increment) is 1.5 (95% CI, 1.3 to 1.9) and Peripheral arterial disease also showed a strong association. The present study showed that rats fed with HFD alone (n = 8) showed a significant increase in serum Hcy at the end of 16 weeks’ treatment, when compared with the normal controls fed a standard diet alone. High-fat diet-fed rats treated with berberine exhibited a significant reduction in serum Hcy by 1.3-fold compared with the high-fat diet treated rats alone. BBR can decrease homocysteine, which indicated that berberine have a beneficial effect in preventing atherosclerosis.

Dyslipidemia is the most important modifiable risk factor of atherosclerosis. The present study demonstrated that administration of berberine along with the high-fat diet effectively reduced serum TC and LDL-c in concordance with other reports [6-11]. Plasma cholesterol level is determined by the net balance between the input of cholesterol into plasma (biosynthesis) and removal rate from plasma [17,18]. The former is mainly dependent on the availability of the rate-limiting enzyme, HMG-CoA reductase (HMGR). High level of hepatic *LDLR* mRNA is associated with improved clearance of plasma LDL-c [17]. Apolipoproteins are carrier proteins that bind lipids to form lipoprotein particles and also serve as enzyme cofactors and lipid transfer carriers that regulate the metabolism of lipoprotein. Thus, the removal rate from plasma in part is related to the level of LDLR and apolipoproteins. We measured mRNA of lipoprotein receptors, apolipoproteins and HMGR in the liver of rats after chronic treatment with berberine. We observed that administration of berbeine significantly upregulated LDLR and apoE gene expression in the liver of rats fed with a high-fat diet. On the contrary, elevated gene expression of hepatic HMGR in long-term HFD-fed rats was down-regulated upon treatment of berberine. Apolipoprotein E (apoE) is synthesized by the liver that mediates the transport and uptake of cholesterol and lipid by way of its high affinity interaction with different cellular receptors, including the low-density lipoprotein (LDL) receptor[19]. One study showed that apoE deficiency causes high serum cholesterol and triglyceride levels and leads to premature artherosclerosis [20]. The previous study indicated that BBR reduces serum cholesterol, LDL-cholesterol via elevating hepatic *LDLR* gene expression through a post-transcriptional mechanism that stabilizes its mRNA [7]. Thus, BBR might increase more clearance of serum LDL-c through its action on LDLR and apoE and reduce cholesterol biosynthesis by suppressing HMGR gene expression.

ApoB is the primary apolipoproteins of chylomicrons and low-density lipoproteins. While it is unclear exactly what functional role apoB plays in LDL, it is the primary apolipoprotein component and is absolutely required for its formation. Lipids are normally exported from the liver in very-low-density lipoproteins (VLDLs), which are complex lipoprotein particles that involve the fusion of a newly synthesized apolipoprotein B-100 (apoB-100) molecule with a triglyceride droplet through the action of microsomal triglyceride transfer protein (MTTP) [21]. A reduction in MTTP function and apoB synthesis and secretion may impair hepatic lipid export and favour triglyceride accumulation in the liver [22,23]. In our previous study [11], long-term high-fat-diet induced significant fat accumulation in rat liver via inhibiting the assembly and secretion of VLDL due to reduction in MTTP function. In this study, apoB gene expression was down-regulated in the liver of rats fed with long-term high-fat-diet compared with normal control rats, but berberine did not affect it. Thus, these suggested that long-term high-fat diet could simultaneously reduce apoB synthesis to favor fat accumulation in the liver, which needs further investigation.

Meanwhile, apoAI mRNA level was decreased in the liver of rats fed with long-term high-fat-diet compared with normal control rats, but berberine also did not affect it.

Atherosclerosis is a systemic inflammatory disease which is associated with several genetic and environmental risk factors. Epidemiologic, clinical and experimental studies show that Long-term intake of high-calories food leads to metabolic derangements characterized by hyperlipidemia and hyperglycemia, which induced the development of atherosclerosis. Thus, SD rats fed with a high-fat diet is a well-established animal model of reproducing the risk factors of premature atherosclerosis development in human [24-27].

Atherosclerosis/angiostasis partially stems from the injury or phenotypic alteration of endothelial cells (ECs), the cells in the frontline against vascular disturbances [28]. The histological examination of HHcy rats revealed an increased recruitment of monocytes to aortic endothelium accompanied by elevated immunostaining for monocyte chemoattractant protein-1, vascular cell adhesion molecule-1 and E-selectin [29]. The present study showed that no abnormal fatty deposits in the intima of arteries were observed and the intima of aorta remained intact in rats fed with a long-term high-fat diet after administration of berberine by gavage for 16 weeks. So berberine could prevent the aortic lesion, which may partially be through above-mentioned mechanism.

Although morphological results also showed no atherosclerotic lesions developed in control group, berberine can improve serum homocysteine and several typical risk factors associated with atherosclerosis, and there is not atherosclerotic lesions observed in berberine-treated group. These results at least suggest that berberine does not promote atherosclerosis development in SD rats fed with long-term high-fat diet.

In contrast to the present results, a recent study showed that berberine can induce foam cell formation and promotes atherosclerosis development in apolipoprotein E-deficient (apoE-/-) mice, which could counter-balance the beneficial effect of lowering serum cholesterol [12]. And the apoE-/- mice are gene-deficient animal model, in which targeted deletion of apoE gene leads to severe hypercholesterolemia and spontaneous. The apoE-/- model has been used widely due to the rapid development of atherosclerosis, despite considerable limitations. A major fault is that plasma cholesterol level is about 8 mmol/L on chow diet, compared with 2 mmol/L for the parent C57Bl/6 mouse, and can increase >70 mmol/L on a high-fat, high-cholesterol (HFC) diet [30]. Another main shortcoming is that most plasma cholesterol is confined to VLDL and not to LDL articles as in humans. Therefore, the different results of these studies are mainly due to the animal model. Our present study at least demonstrated that berberine did not increase the risk of atherosclerosis in SD rats fed with a long-term high-fat diet, although these rats are not the typical model of atherosclerosis.

In summary, the present study has demonstrated that berberine can decrease serum homocysteine and several traditional risk factors associated with atherosclerosis via regulating LDLR, apoE and HMGR gene expression in rats fed with high-fat diet. Our findings illustrate that berberine may represents a promising agent for the pristine prevention of atherosclerosis.

## Methods

Berberine, serum triglyceride determination Kit, and LDL-c Kit were purchased from Sigma-Aldrich, St. Louis, MO.

### Animal experiment

The animal component of this study has been described in detail in our recently published data [11]. Briefly, healthy male Sprague –Dawley (SD) rats [Grade SPF, certificate number of the breeder: SCXK(Hu) 2007-0005] weighing 190-210 g from the Animal Development Center (Chinese Academy of Sciences, Shanghai, China) were given free access to food and water and were maintained on a 12/12-hour light/dark cycle. Rats received either a regular rodent chow (normal diet: 62.3% carbohydrate/12.5% fat/24.3% protein calories) or a high-fat diet (32.6% carbohydrate/51.0% fat/16.4% protein calories) for 24 weeks. After 8 weeks of feeding, rats on the HFD received randomly intragastric injection of either berberine (200 mg · kg^-1^· day^-1^) or vehicle for 16 weeks. Rats fed the normal diet received the equal volume of vehicle (0.5% methylcellulose, N group) as a control group. After an overnight fasting period, blood samples were also collected for measurement of serum Hcy, TC and LDL-c levels using commercially available kits. The liver tissues were stored in liquid nitrogen for biochemical and molecular analysis.

All experimental procedures involving the use of animals were conducted in conformity with PHS policy and were approved by the Animal Use and Care Committee of Fudan University.

### Measurement of serum hcy by ELISA

Serum homocysteine ELISA kit was from Maibiotechnology, China. Add 100μl each of dilutions of standard, blank and samples into the appropriate wells. Mix well and incubate for 2 hours at 37°C. Remove the liquid of each well, and wash with 400 of 1X Wash Solution to each well. Then add 100μl of biotinylated antibody working solution to each well. After incubate for 1 hour, washing the wells. Add 100μl of enzyme conjugate working solution to each well. Incubate for 30 minutes at 37°C after covering it with the Plate sealer. Aspirate the solution and wash with 400μl of 1X Wash Solution to each well. Then add 100μl of TMB solution working solution to each well. Incubate for 15 minutes at 37°C after covering it with the Plate sealer. Protect from light. Add 50μl of Stop Solution to each well. Then, run the microplate reader and conduct measurement at 450 nm immediately.

### Measurement of serum TC

Amplex red cholesterol assay kit (A12216) was obtained from invitrogen detection technologies. Prepare a cholesterol standard curve. Dilute serum in 1XReaction Buffer. Pipet 50μl of the diluted samples and controls into separate wells of a microplate. Begin the reactions by adding 50μl of the Amplex Red reagent/HRP/cholesterol oxidase/cholesterol esterase working solution to each microplate well containing the samples and controls. Incubate the reactions for 30minutes at 37°C, protected from light. Measure the fluorescence in a fluorescence microplate reader using excitation in the range of 530-560 nm and emission detection at ~ 590 nm. According to the standard curve, get the cholesterol level of each sample.

### Measurement of serum LDL-c

Prepare working solution containing cholesterol oxidase/cholesterol esterase/catalase/peroxidase. Pipet 5μl of the diluted samples and controls into separate wells of a microplate. Then add 500μl of working solution to each microplate well. Mix well and incubate the reactions in the 37°C water bath for 10 minutes. Measure the absorbance value at 546 nm.

### Hepatic total cholesterol level

Hepatic lipids were extracted according to the method of Folch et al. [31]. Briefly, lipid was extracted from frozen liver tissues (30 mg) by homogenization in 1 ml of 2:1 chloroform: methanol, followed by shaking at room temperature for overnight and centrifugation at 3000 rpm for 10 min. Aliquots (400 l) of the organic-extract lipid suspension were analyzed for hepatic total cholesterol concentration using commercial diagnostic kits. Hepatic TC content was defined as mg of cholesterol per gram of the liver.

### Histological analysis

After the rats (n = 8 pre group) were sacrificed, the aortas were removed from the region of the proximal aorta and fixed in phosphate-buffered 10% formalin, which was subsequently stained with hematoxylin and eosin (HE). The aorta was then divided into 2 sections, one of which was embedded in paraffin blocks and the other in O.C.T. compound. A section from each paraffin block was stained with HE to examine the pathologic structures of the aorta and serial cryosections (five consecutive slices each sample) were stained with Sudan Ш to evaluate aortic lesion. These pathological slices were observed by the experienced pathologists in blinded state.

### Real-time quantitative PCR (qPCR) analysis

Total RNA was isolated from liver tissues using Trizol reagent (Invitrogen, Carlsbad, CA, USA). cDNA was synthesized by reverse transcription using ReverTra Ace (Toyobo, Osaka, Japan). The SYBR Green PCR Master Mix (Toyobo, Osaka, Japan) was used for qPCR with a sequence detection system (ABI PRISM7900, Applied Biosystems, Foster City, CA, USA). EightμL reaction mixture contained 1μL of cDNA and 125 nmol/L of primers. The specific primers of PCR amplification are described in Table 1. The same reaction was performed in triplicate with The relative gene expression was calculated using the 2^−ΔΔCt^ as described previously [32].

**Table 1:** Primer sequences for real-time quantitative PCR

**Gene**	**Forward primer (5’- 3’)**	**Reverse primer (5’- 3’)**
LDLR	CCAGTGCGGCGTAGGATT	GGGACTCATCGGAGCCAT
HMGR	AGAATATAGCGCGTGGGATG	GACATACAGCCAAAGCAGCA
apoB	TAAATGGAGCACTTTTCAAG	GGAACAGCAGCAGTAGCG
apoAI	GCCACTGTGTATGTGGATGC	AACCCAGAGTGTCCCAGTTG
apoE	AGGAGCAGACCCAGCAGATA	GGAGTTGGTAGCCACAGAGG
SR	GCCACTGGTCCTTGTTTGTT	TGGGAGCGCTGACTTTTACT
beta-actin	GATTACTGCCCTGGCTCCTA	TCATCGTACTCCTGCTTGCT

### Statistical analysis

All data were presented as mean ± SEM. Significance was assessed by Sudent’s *t*-test. All *p* values were two-tailed and *p* values of less than 0.05 were considered to be statistically significant.

## Abbreviations

BBR: Berberine; SD: Sprague –Dawley; HFD: High-fat diet; Hcy: Homocyteine; TC: Total cholesterol; LDL-c: Low density lipoprotein cholesterol; HE: Hematoxylin and eosin; HMGR: 3-hydroxy-3-methyl-glutaryl-CoA reductase; LDLR: LDL receptor; apoE: Apolipoprotein E; SR: Scavenger receptor; HHcy: Hyperhomocysteine; apoE-/-: Apolipoprotein E-deficient; tHcy: Total Hcy; CAD: Coronary artery disease; VLDL: Very low density lipoprotein; MTTP: Microsomal triglyceride transfer protein; ECs: Endothelial cells; HFC: High-fat: high-cholesterol; qPCR: Real-time quantitative PCR.

## Competing interests

The authors declare that they have no competing interests.

## Authors’ contributions

XG designed the research and revised this manuscript; XC and HY carried out animal experiment and wrote the paper; QX performed parts of western immunoblot analysis and helped to draft the manuscript; HB and MX participated in parts of data analysis; TZ performed Histological analysis. All authors read and approved the final manuscript.
